# Whole-Genome Sequencing Identifies *PPARGC1A* as a Putative Modifier of Cancer Risk in *BRCA1/2* Mutation Carriers

**DOI:** 10.3390/cancers14102350

**Published:** 2022-05-10

**Authors:** Qianqian Zhu, Jie Wang, Han Yu, Qiang Hu, Nicholas W. Bateman, Mark Long, Spencer Rosario, Emily Schultz, Clifton L. Dalgard, Matthew D. Wilkerson, Gauthaman Sukumar, Ruea-Yea Huang, Jasmine Kaur, Shashikant B. Lele, Emese Zsiros, Jeannine Villella, Amit Lugade, Kirsten Moysich, Thomas P. Conrads, George L. Maxwell, Kunle Odunsi

**Affiliations:** 1 Department of Biostatistics & Bioinformatics, Roswell Park Comprehensive Cancer Center, Buffalo, NY 14263, USA; jie.wang@roswellpark.org (J.W.); han.yu@roswellpark.org (H.Y.); qiang.hu@roswellpark.org (Q.H.); mark.long@roswellpark.org (M.L.); spencer.rosario@roswellpark.org (S.R.); emily.schiller@roswellpark.org (E.S.); 2 Gynecologic Cancer Center of Excellence, Department of Gynecologic Surgery and Obstetrics, Uniformed Services University and Walter Reed National Military Medical Center, 8901 Wisconsin Avenue, Bethesda, MD 20889, USA; batemann@whirc.org (N.W.B.); conrads@whirc.org (T.P.C.); george.maxwell@inova.org (G.L.M.); 3 Henry M. Jackson Foundation for the Advancement of Military Medicine, Inc., 6720A Rockledge Dr., Suite 100, Bethesda, MD 20817, USA; gauthaman.sukumar.ctr@usuhs.edu; 4 The American Genome Center, Uniformed Services University of the Health Sciences, Bethesda, MD 20814, USA; clifton.dalgard@usuhs.edu (C.L.D.); matthew.wilkerson@usuhs.edu (M.D.W.); 5 Department of Anatomy Physiology and Genetics, Uniformed Services University, 4301 Jones Bridge Road, Bethesda, MD 20814, USA; 6 Center for Immunotherapy, Roswell Park Comprehensive Cancer Center, Buffalo, NY 14263, USA; raya.huang@roswellpark.org (R.-Y.H.); amit.lugade@roswellpark.org (A.L.); 7 Department of Gynecologic Oncology, Roswell Park Comprehensive Cancer Center, Buffalo, NY 14263, USA; jasmine.kaur@roswellpark.org (J.K.); shashi.lele@roswellpark.org (S.B.L.); emese.zsiros@roswellpark.org (E.Z.); 8 Division of Gynecologic Oncology, Lenox Hill Hospital/Northwell Health Cancer Institute, Donald and Barbara Zucker School of Medicine at Hofstra/Northwell, New York, NY 11549, USA; jvillella@northwell.edu; 9 Department of Cancer Prevention and Control, Roswell Park Comprehensive Cancer Center, Buffalo, NY 14263, USA; moysich@roswellpark.org; 10 Women’s Health Integrated Research Center, Women’s Service Line, Inova Health System, 3289 Woodburn Rd, Annandale, VA 22003, USA; 11 Department of Obstetrics and Gynecology, University of Chicago, Chicago, IL 60637, USA; 12 University of Chicago Medicine Comprehensive Cancer Center, Chicago, IL 60637, USA

**Keywords:** *BRCA* modifier, cancer susceptibility gene, whole-genome sequencing, ovarian cancer, breast cancer

## Abstract

**Simple Summary:**

In search of genetic factors that affect cancer risks in *BRCA* carriers, we carried out the first whole-genome sequencing study in a unique registry of familial ovarian cancer, selected to enrich with *BRCA1/2* carriers. We are the first to survey rare variants, particularly the non-coding variants for *BRCA* modifier genes and identified *PPARGC1A*, a master regulator of mitochondrial biogenesis, as a novel putative *BRCA* modifier. This finding can help improve cancer risk prediction and provide personalized preventive care for *BRCA* carriers.

**Abstract:**

While *BRCA1* and *BRCA2* mutations are known to confer the largest risk of breast cancer and ovarian cancer, the incomplete penetrance of the mutations and the substantial variability in age at cancer onset among carriers suggest additional factors modifying the risk of cancer in *BRCA1/2* mutation carriers. To identify genetic modifiers of *BRCA1/2*, we carried out a whole-genome sequencing study of 66 ovarian cancer patients that were enriched with *BRCA* carriers, followed by validation using data from the Pan-Cancer Analysis of Whole Genomes Consortium. We found *PPARGC1A*, a master regulator of mitochondrial biogenesis and function, to be highly mutated in *BRCA* carriers, and patients with both *PPARGC1A* and *BRCA1/2* mutations were diagnosed with breast or ovarian cancer at significantly younger ages, while the mutation status of each gene alone did not significantly associate with age of onset. Our study suggests *PPARGC1A* as a possible *BRCA* modifier gene. Upon further validation, this finding can help improve cancer risk prediction and provide personalized preventive care for *BRCA* carriers.

## 1. Introduction

*BRCA1* and *BRCA2* are the two most well-known cancer predisposition genes. Inheritance of a *BRCA1* or *BRCA2* mutation greatly increases lifetime risk of breast cancer and ovarian cancer [1,2]. While lifetime risk of developing ovarian cancer for women in the general population is about 1.2% [3], the risk was estimated to be 39–59% and 11–17% for women who carry *BRCA1* and *BRCA2* mutations, respectively, by age 70–80 [4,5,6,7]. Preventive strategies have therefore been implemented to reduce cancer risk in *BRCA1/2* carriers [8,9,10]. On the other hand, the penetrance of *BRCA1/2* mutations is not complete, and there is substantial variability in age of cancer onset among carriers. These observations support the hypothesis that cancer risks in *BRCA1/2* mutation carriers are modified by other factors. Over the past two decades, significant efforts have been invested in search of these modifying factors, among the most influential of which is the Consortium of Investigators of Modifiers of *BRCA1/2* (CIMBA) [11]. These efforts resulted in the discovery of a number of risk modifiers for *BRCA1/2* mutations, including environmental, reproductive, and genetic factors [12,13,14,15].

To date, studies to identify genetic modifiers of *BRCA1/2* were mainly carried out in three ways: candidate gene studies, investigation of specific variants discovered by genome-wide association studies (GWAS) to associate with cancer risks in the general population, and GWAS carried out specifically in *BRCA1* or *BRCA2* carriers [13,16,17,18,19]. The potential *BRCA1/2* genetic modifiers identified in these studies are all common variants with relatively small effect sizes. On the other hand, the contribution of rare variants, which are more likely to have large effect sizes and/or direct functional consequences, in modifying cancer risks of *BRCA1/2* carriers has not been systematically explored. Therefore, we employed whole-genome sequencing (WGS) technology to identify genetic modifiers of *BRCA1/2* that are driven by rare variants.

To maximize the likelihood of discovering genetic modifiers of *BRCA1/2*, we performed WGS on a total of 66 ovarian cancer (OC) patients that were enriched with *BRCA* carriers. A total of 49 of these patients were selected from the Familial Ovarian Cancer Registry (FOCR) [20,21] to have a strong family history of OC. The discovered candidates were evaluated using independent WGS data of 247 ovarian and breast cancer patients from the Pan-Cancer Analysis of Whole Genomes (PCAWG) Consortium [22]. To our knowledge, this is the first and largest WGS study of *BRCA1/2* modifiers to date, and we report *PPARGC1A* as a novel putative genetic modifier of ovarian and breast cancer risk for *BRCA1/2* carriers.

## 2. Materials and Methods

### 2.1. Study Population

A total of 50 hereditary OC patients from 48 families were selected from FOCR (formerly known as the Gilda Familial Ovarian Cancer Registry) for WGS based on DNA availability, prior genetic test results of *BRCA1/2*, and strong family history. The FOCR housed at Roswell Park Comprehensive Cancer Center (RPCCC) recruits families with two or more cases of OC, families with three or more cases of cancer on the same side of family with at least one being OC, families with at least one female having two or more primary cancers and one of the primaries being OC, and families with two or more cases of cancer with at least one being OC diagnosed at an early age of onset (45 years old or younger) [21]. Families provide written informed consent under an institutional protocol CIC95-27. Cases are verified by medical record and/or death certificate when required, and a registry pathologist verifies stage and histology. The registry comprises 50,401 individuals including 5614 ovarian cancers from 2636 unique families. A total of 27 of the 50 FOCR patients were known carriers of *BRCA* from prior genetic testing performed by Myriad or inhouse [21]. An additional 18 RPCCC patients with sporadic OC were also included for WGS.

### 2.2. Whole-Genome Sequencing, Variant Calling, and Variant Filtering

Sequencing library preparation and whole-genome sequencing was performed at The American Genome Center at the Uniformed Services University, Bethesda, MD (Supplementary Methods). The GATK data pre-processing workflow was used to generate analysis-ready alignments (Supplementary Methods). DeepVariant (v0.5) was used to call single nucleotide variants (SNVs) and small insertions and deletions (indels) in standard VCF format for each sample with a convolutional neural network model [23]. Structural variants (SV) were detected using a structural variant calling workflow developed by bcbio, which used an integrative caller MetaSV [24] that combines the results from four separate methods, CNVkit [25], Manta [26], LUMPY [27], and Wham [28]. We only considered SVs longer than 50 bp and required SVs to be detected by at least two of the four methods with ≥3 supporting reads (split read or disconcordant read) in each method. Using the genotypes of SNVs, we performed sample level quality assessment using the Bioconductor package SeqSQC [29]. One FOCR patient and one sporadic OC patient were identified as population outliers and hence were excluded from further analyses.

A series of filters were applied to keep only rare and functional variants in our analysis (Supplementary Methods). Variants in the eight genes with significantly higher mutation rate in *BRCA* carriers of our WGS discovery cohort (Table 1) were manually inspected to ensure reliable variant calls.

### 2.3. Statistical Analysis

One-sided Fisher’s exact test was used to test whether the gene mutation frequency is higher in *BRCA* carriers than non-carriers.

### 2.4. Network Propagation and Pathway Enrichment Analysis

Using HotNet2 [30] and the Reactome functional interaction networks [31], we applied a network propagation analysis on the −log10 scores of the one-sided Fisher’s exact test *p*-values calculated by comparing gene mutation frequencies between *BRCA* carriers and non-carriers within the 49 hereditary OC patients. Only genes with Fisher’s exact test *p*-values ≤ 0.6 were included in the analysis. The statistical significance of the identified sub-networks was based on the number and size of the identified sub-networks compared to those found using a permutation test. We used 100 permutations and a minimum network size of 2 for statistical testing.

To examine the biological functions of each significant gene sub-network, pathway enrichment analysis of genes in each sub-network was performed using hypergeometric testing based on the Reactome pathway database [31]. Multiple testing was corrected using the Benjamini–Hochberg method. For each sub-network, pathways with adjusted *p*-values < 0.05 were considered significantly enriched.

### 2.5. Validation Using PCAWG Breast and Ovarian Cancer Cohorts

We obtained germline genetic variants called by PCAWG and kept only breast and ovarian cancer patients that were of European ancestry. Sample level quality assessment was performed using the Bioconductor package SeqSQC [29], which resulted in the removal of nine problematic samples. Germline variants of the remaining PCAWG samples were filtered and annotated in the same way as described above for the germline variants in our WGS data. We kept only the variants whose target genes were *BRCA1*, *BRCA2*, *PPARGC1A*, and *PBX1* in our analysis.

### 2.6. Study Approval

The study was approved by Institutional Review Boards. All participants provided written informed consent.

## 3. Results

### 3.1. Study Population in the Discovery Stage

**Figure 1:**
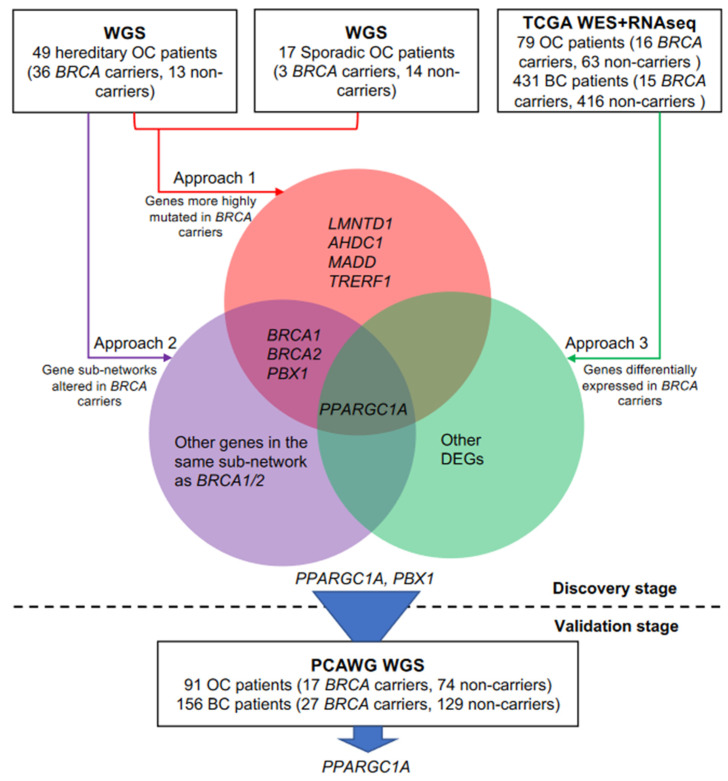
Schema of the analyses to identify genetic modifiers of ovarian and breast cancer risks in *BRCA* carriers.

Our discovery WGS cohort consisted of 49 OC patients from the FOCR (formerly known as the Gilda Familial Ovarian Cancer Registry) with a strong family history of OC [20,21] and 17 sporadic OC patients (Figure 1, Tables S1–S3); all were of European descent. Among the 49 hereditary OC patients, 27 were known *BRCA* carriers from prior genetic testing and were purposely included to enrich for *BRCA* carriers in our discovery cohort (Materials and Methods).

### 3.2. Discovery of Candidate Genes That Modify Cancer Risks of BRCA1/2

The germline genetic variants were detected from the discovery WGS data using a deep learning variant calling algorithm [23], which has been shown to achieve higher sensitivity and specificity in pathogenic variant detection than standard methods [32]. A series of stringent variant filtering steps were carried out to retain only rare (MAF < 0.5% in European population) and functional variants for subsequent analyses (Materials and Methods). The 27 known BRCA carriers were confirmed to carry *BRCA1/2* mutations from our WGS analysis, and 12 more patients in the discovery cohort were found to be *BRCA* carriers, including 9 hereditary OC patients and 3 sporadic OC patients.

To identify *BRCA* modifiers that increase cancer risks in *BRCA* carriers, we utilized three different approaches. First, we compared mutation frequency of each gene between *BRCA* carriers and non-carriers in our discovery cohort using Fisher’s exact test and focused on genes that are more frequently mutated in *BRCA* carriers (Table 1 and Table S4), requiring uncorrected Fisher’s exact test *p*-value ≤ 0.05 in consideration of our relatively small sample size. In addition, we also required the genes to have significantly higher mutation frequency in *BRCA* carriers in the analysis of the 49 hereditary OC patients, assuming the effect of *BRCA* modifier is most enriched in cancer patients with family history. Eight genes satisfied both criteria, including *BRCA1/2* and a known cancer gene *PBX1*.

**Table 1:** Comparison of gene mutation frequency between *BRCA* carriers and non-carriers in the discovery cohort.

Gene	Hereditary OC (36 *BRCA* Carriers vs. 13 Non-Carriers)					Hereditary OC + Sporadic OC (39 *BRCA* Carriers vs. 27 Non-Carriers)				
	# and Fraction of Mutated *BRCA* Carriers		# and Fraction of Mutated Non-Carriers		*p*-Value *	# and Fraction of Mutated *BRCA* Carriers		# and Fraction of Mutated Non-Carriers		*p*-Value *
*BRCA1*	29	0.81	0	0.00	2.95 × 10^−7^	30	0.77	0	0.00	3.84 × 10^−11^
*BRCA2*	10	0.28	0	0.00	3.09 × 10^−2^	12	0.31	0	0.00	7.94 × 10^−4^
*PPARGC1A*	11	0.31	0	0.00	2.06 × 10^−2^	11	0.28	1	0.04	9.99 × 10^−3^
*PBX1*	14	0.39	1	0.08	3.49 × 10^−2^	14	0.36	3	0.11	2.15 × 10^−2^
*LMNTD1*	9	0.25	0	0.00	4.58 × 10^−2^	9	0.23	0	0.00	5.73 × 10^−3^
*AHDC1*	9	0.25	0	0.00	4.58 × 10^−2^	9	0.23	1	0.04	3.01 × 10^−2^
*MADD*	9	0.25	0	0.00	4.58 × 10^−2^	9	0.23	1	0.04	3.01 × 10^−2^
*TRERF1*	9	0.25	0	0.00	4.58 × 10^−2^	10	0.26	1	0.04	1.75 × 10^−2^

* Raw *p*-value from one-sided Fisher’s exact test (H_a_: gene mutation frequency in *BRCA* carriers ≥ the frequency in non-carriers).

**Figure 2:**
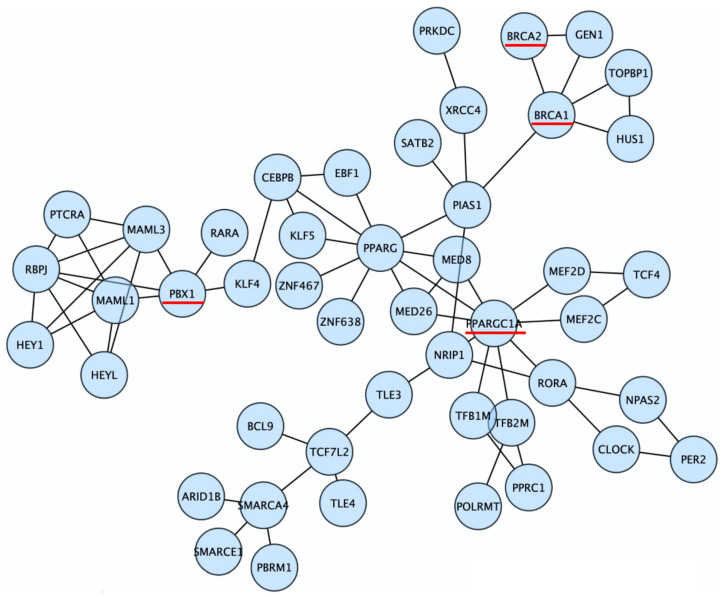
The largest gene sub-network significantly altered in *BRCA* carriers. Genes that were significantly highly mutated in *BRCA* carriers (Table 1) were highlighted by red underscores.

Next, we adopted a network-based approach to identify the pathways that are altered in *BRCA* carriers based on the hypothesis that malfunction of certain biological processes increases cancer risk in *BRCA* carriers, and within those processes, multiple genes instead of a unique gene can be targeted by germline genetic mutations. Specifically, we mapped genes that showed elevated mutation rates in *BRCA* carriers within the hereditary OC cohort onto Reactome functional interaction networks [31] and used the network propagation method HotNet2 [30] to detect sub-networks that contain multiple contributing neighboring genes (Materials and Methods). A total of 15 significant sub-networks were identified (HotNet2 permutation *p*-value = 0.01). The largest sub-network contained *BRCA1/2* and 45 other genes (Figure 2 and Figure S1). The biological pathways that were significantly enriched in this sub-network included transcriptional regulation of white adipocyte differentiation, transcriptional regulation by Notch3 and Notch1, circadian clock, transcriptional activation of mitochondrial biogenesis, and SUMOylation (Table S5). Assuming the *BRCA* modifiers alter the same biological processes as *BRCA1/2*, we focused on the 47 genes within the same sub-network as *BRCA1/2* in further analysis.

Finally, under our prior hypothesis that any *BRCA* modifier gene would be more highly mutated in *BRCA* carriers, we would expect the expression of any such modifier gene to differ between *BRCA* carriers and non-carriers, assuming the genetic mutation affects its gene expression. Therefore, we utilized both RNAseq and whole-exome sequencing (WES) data from The Cancer Genome Atlas (TCGA) Pan-Cancer analysis project to detect differentially expressed genes (DEGs) between *BRCA* carriers and non-carriers in breast or ovarian cancer (Supplementary Methods). A total of 4600 DEGs were identified in breast cancer by comparing 15 *BRCA* carriers with 416 non-carriers, while 189 DEGs were identified in OC by comparing 16 *BRCA* carriers with 63 non-carriers. Among these DEGs, only one, *PPARGC1A*, was identified from our WGS analysis as more highly mutated in *BRCA* carriers than non-carriers. It was significantly up-regulated in *BRCA* carriers of breast cancer (log_2_ fold change (carriers vs. non-carriers) = 1.86, adjusted *p* = 6.10 × 10^−3^).

### 3.3. Independent Validation of BRCA Modifier Candidate Genes

Using the three different approaches described above, we found two potential *BRCA* modifier candidates, *PPARGC1A* and *PBX1*, which were identified by three and two approaches, respectively, (Figure 1). We then sought to validate these two genes using independent WGS cohorts of breast and ovarian cancer from the Pan-Cancer Analysis of Whole Genomes (PCAWG) Consortium [22], consisting of both the TCGA and the International Cancer Genome Consortium (ICGC). Among the 156 and 91 breast cancer and OC patients from PCAWG, only 17% and 19% were *BRCA* carriers, respectively, which was similar to the value in the sporadic OC cases of our discovery cohort but much lower than in the hereditary OC cases of our discovery cohort, where 73% were *BRCA* carriers.

**Figure 3:**
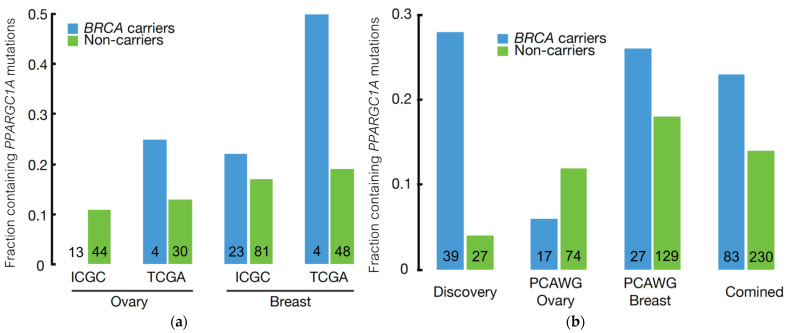
Fraction of *BRCA* carriers and non-carriers that contained *PPARGC1A* mutations: (**a**) analysis within the PCAWG validation cohorts; (**b**) analysis within our discovery cohort, the PCAWG validation cohorts, and all cohorts combined. The numbers inside the bars are the numbers of *BRCA* carriers and non-carriers.

In PCAWG’s breast cancer cohort, we observed a trend of higher *PPARGC1A* mutation frequency in *BRCA* carriers than non-carriers (Figure 3a, Table S6). This trend was also found in PCAWG’s ovarian cancer cases from TCGA, but not in those from ICGC (Table S6). When combining the discovery and validation cohorts, the *PPARGC1A* gene in *BRCA* carriers possessed more deleterious mutations than in non-carriers with borderline significance (*p* = 0.055, Figure 3b). The other candidate gene, *PBX1,* did not show a higher mutation rate in *BRCA* carriers in either PCAWG breast or ovarian cancer cohort (Table S7).

### 3.4. PPARGC1A as a Novel BRCA Modifier Candidate Gene

**Figure 4:**
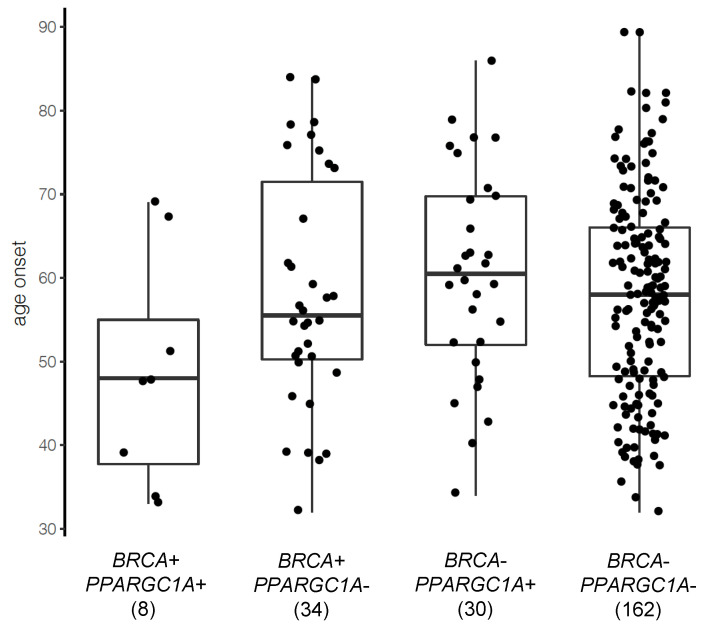
The distribution of cancer age onset by *BRCA* and *PPARGC1A* mutation status in PCAWG breast and ovarian cancer patients. The number in parenthesis is the sample size of each group.

We then investigated whether *PPARGC1A* mutations affect age of onset of breast and ovarian cancer in the PCAWG patients. We found that the interaction between *PPARGC1A* mutation status and *BRCA* status significantly associated with an earlier age of cancer onset (*p* = 0.03), while the main effect of each gene alone was not significant (*p* = 0.33 and 0.96, respectively, for *PPARGC1A* status and *BRCA* status). Patients who carried both *BRCA1/2* mutations and *PPARGC1A* mutations were diagnosed with breast or ovarian cancer at a significantly younger age (Figure 4). The median age of onset was 48, 55.5, 60.5, and 58 respectively for patients carrying mutations in both *PPARGC1A* and *BRCA1/2* genes, in *BRCA1/2* genes only, in *PPARGC1A* only, or in none of the three genes. The interaction term remained significant when restricting on breast cancer patients, but not in a regression model on the smaller cohort of ovarian cancer patients, where only one patient carried both *BRCA* and *PPARGC1A* mutations (Table S8). Consistent patterns were observed when *BRCA1* and *BRCA2* status were included in the regression model (Table S9).

**Figure 5:**
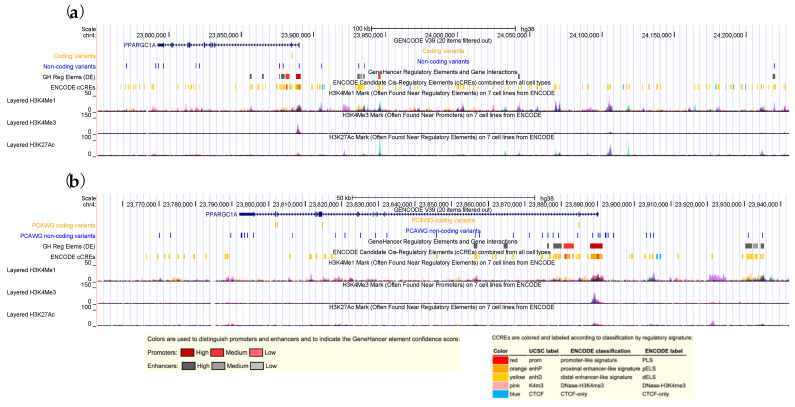
The *PPARGC1A* variants identified in the discovery stage (**a**); and validation stage (**b**); respectively.

The majority of the *PPARGC1A* mutations we identified in both the discovery and the validation cohorts were non-coding variants (Figure 5). To investigate how these non-coding variants affect *PPARGC1A* expression, we compared *PPARGC1A* expression between the carriers of non-coding variants and the non-carriers using PCAWG RNA-seq data [33] and observed a trend of lower expression in the carriers (*p* = 0.09 and 0.44 for ovarian cancer and breast cancer, respectively, Figure S2).

## 4. Discussion

In this first WGS study to discover putative *BRCA* genetic modifiers, we performed WGS on 66 OC patients that were enriched with *BRCA* carriers and identified two genes, *PPARGC1A* and *PBX1*, to be highly mutated in *BRCA* carriers and within the same gene subnetwork with *BRCA1/2*. In addition, *PPARGC1A* was found to be differentially expressed between *BRCA* carriers and non-carriers within the TCGA breast cancer cohort. Our independent validation in PCAWG observed a similar trend of a higher *PPARGC1A* mutation rate in *BRCA* carriers than non-carriers for patients with breast cancer. Importantly, we found that patients with both *PPARGC1A* and *BRCA1/2* mutations were diagnosed with breast or ovarian cancer at a significantly younger age, while the effect of each gene alone was not significant. Therefore, our results suggest *PPARGC1A* to be a potential new *BRCA* modifier. While previous candidate gene studies of common genetic variants linked *PPARGC1A* with ovarian cancer and familial breast cancer risk [34,35], our WGS study, focusing on rare and functional variants, was the first to reveal the effect of *PPARGC1A* in the context of *BRCA1/2* mutation. The *PPARGC1A* mutations we identified through WGS were dominantly non-coding variants, which highlights the importance of going beyond just the gene coding regions in search of *BRCA* modifiers and cancer predisposition genes. It is worth noting that the pathways involving *PPARGC1A*, such as transcriptional regulation of white adipocyte differentiation and transcriptional activation of mitochondrial biogenesis, were found to be disturbed in *BRCA* carriers (Table S3). Future studies targeting these pathways may allow identification of additional *BRCA* modifiers.

PPARGC1A, also known as Peroxisome proliferator-activated receptor gamma coactivator 1-alpha (PGC-1α), is a coactivator of PPARγ, which is a crucial gene regulating *BRCA1* gene expression [36]. In addition, PPARGC1A is a master regulator of mitochondrial biogenesis and function. It is essential for cancer cells to rapidly adapt to energy-demanding situations. Both increased and decreased PPARGC1A expression have been reported in a range of cancer types and associated with a worse prognosis [37,38,39]. These contradicting observations are now thought to result from cancer cells exploiting PPARGC1A to provide them metabolic plasticity to support their evolving needs along the course of cancer development [37,38,40,41]. During early tumorigenesis, PPARGC1A may be downregulated to facilitate the increased consumption of glucose and glutamine in cancer cells [37,40]. This is consistent with our findings of significantly earlier cancer development in carriers with mutations in both *PPARGC1A* and *BRCA1/2,* as well as lower PPARGC1A expression in the *PPARGC1A* mutation carriers. On the other hand, a recent study demonstrated inhibition of PPARGC1A in tumor infiltrating T cells leads to T cell exhaustion and tumor immune escape [42]. *BRCA1* and *BRCA2* are key homologous recombination genes. Defects in *BRCA1/2* result in tumors with extensive genomic instability that stimulates inflammatory signaling [43,44,45,46,47,48,49]. Therefore, cancer cells that are genomically unstable must evolve to escape immune surveillance in order to avoid being cleared by the immune system [43]. Because tumor-infiltrating T cells in *PPARGC1A* mutation carriers experience *PPARGC1A* inhibition and T cell exhaustion due to metabolic insufficiency [42], *BRCA1/2*-mutant cancer cells in *PPARGC1A* mutation carriers have an advantage in escaping immune surveillance and thus can develop into tumors earlier than in individuals without *PPARGC1A* mutations. In summary, loss of PPARGC1A due to germline *PPARGC1A* variants might stimulate tumor development by giving tumor cells a metabolic advantage or weakening immune surveillance, or both.

A major strength of our study is the enrichment for *BRCA* carriers from a familial ovarian cancer registry. However, there are limitations to our study. While we have leveraged the largest publicly available WGS collection of breast and ovarian cancer patients from PCAWG, our validation study is limited by the relatively small sample size. Furthermore, the validation power is reduced due to over-representation of sporadic cancer and the small number of *BRCA* carriers in these publicly available WGS cohorts. A future sequencing study of the entire *PPARGC1A* locus in large ovarian and breast cancer cohorts, particularly in cancer patients with family history, is warranted to further validate our finding of *PPARGC1A* as a *BRCA1/2* genetic modifier. Knocking out *PPARGC1A* in breast or ovarian cancer mouse models with mutated *BRCA1/2* [50,51,52] will also help to investigate the effect of *PPARGC1A* in increasing cancer risk in the context of *BRCA1/2* mutations and to reveal the underlying biological mechanism.

## 5. Conclusions

We conducted the first WGS study of hereditary OC patients enriched with *BRCA* carriers and followed with a validation study using the largest WGS collection of OC and BC patients to date to identify *PPARGC1A* as a possible *BRCA* modifier gene. Given the impact of *PPARGC1A* on the age of onset of OC and BC among *BRCA* mutation carriers, our results could have significant implications for cancer risk prediction and personalized preventive care for *BRCA* carriers. Future follow-up studies including additional sequencing and functional experiments are warranted to confirm these findings.

## Data Availability

Data are restricted due to ethical concerns in keeping with the institute’s policies on germline variation data and the level of patient consent gained. Data are available from the Familial Ovarian Cancer Registry (ovarianregistry@roswellpark.org) for researchers who meet the criteria for access to confidential data. The results published here are in part based upon data generated by The Cancer Genome Atlas (dbGaP Study Accession: phs000178.v10.p8) managed by the NCI and NHGRI. Information about TCGA can be found at http://cancergenome.nih.gov.

## Supplementary Materials

The following supporting information can be downloaded at: https://www.mdpi.com/article/10.3390/cancers14102350/s1, Supplementary Methods; Figure S1: The remaining 14 gene sub-networks significantly altered in *BRCA* carriers. Genes that were significantly highly mutated in *BRCA* carriers (Table 1) are highlighted by red underscores.; Figure S2: *PPARGC1A* expression between carriers of non-coding mutations and non-carriers in PCAWG. Gene expression levels (FPKM-UQ) were estimated using the FPKM metric, based on alignments from the TopHat and STAR algorithms and normalized with the Upper Quartile method. FPKM, fragments per kilobase of transcript per million mapped reads. *p*-values were calculated using one-sided Wilcoxon rank sum test between 11 carriers and 41 non-carriers in breast cancer and between 7 carriers and 60 non-carriers in ovarian cancer; Table S1: Characteristics of the discovery cohort; Table S2: The sequencing coverage and quality statistics of whole-genome-sequenced FOCR patients; Table S3: The sequencing coverage and quality statistics of whole-genome sequenced sporadic OC patients; Table S4: The *PPARGC1A* variants included in our analyses; Table S5: The pathways significantly enriched in the genes within the largest gene sub-network significantly altered in *BRCA* carriers; Table S6: Comparison of *PPARGC1A* mutation frequency between *BRCA* carriers and non-carriers in the validation cohorts; Table S7: Comparison of *PBX1* mutation frequency between *BRCA* carriers and non-carriers in the validation cohorts; Table S8: Linear regression between cancer age onset and gene mutation status (*BRCA*, *PPARGC1A*) in PCAWG; Table S9: Linear regression between cancer age onset and gene mutation status (*BRCA1*, *BRCA2*, *PPARGC1A*) in PCAWG. (References [53,54,55,56,57,58,59,60,61,62,63,64] cited in the supplementary materials)
