# Supplementary material for: Whole-Genome Sequencing Identifies PPARGC1A as a Putative Modifier of Cancer Risk in BRCA1/2 Mutation Carriers
Source: Cancers (Basel). 2022 May 10;14(10):2350. doi: 10.3390/cancers14102350 (PMC9139302; doi:10.3390/cancers14102350)
Supplement: Supplementary file 1 [file cancers-14-02350-s001.zip › cancers-1665457-supplementary.pdf]

Supplementary Methods

Whole-genome sequencing

Genomic DNA (gDNA) was diluted to 20 ng/μL using Resuspension Buffer (RSB, Illumina) and 55 μL were transferred to Covaris microTubes (Covaris, Woburn, MA). The normalized gDNA was then sheared on an LE2200 focused-ultrasonication system (Covaris) to generate DNA fragments with average peak size of 450 bp. Library preparation was conducted using the TruSeq DNA PCR-free Library Preparation Kit (Illumina, San Diego, CA) following manufacturer's instructions with minor modifications for automated liquid handling. The size distribution of the final sequencing libraries was assessed using the Fragment Analyzer (AATI). Libraries were quantified by real-time quantitative PCR, using the KAPA Library Quantification Kit, Complete kit (KAPA Biosystems) optimized for the LightCycler 480 instrument (Roche). Libraries were then normalized to 2 nM and clustered on the Illumina cBot 2 using a HiSeq X HD Paired-End Cluster Generation Kit v2. Paired-end sequencing was performed with the HiSeq X HD SBS Kit (300 cycles) on the Illumina HiSeq X. FASTQ files were created from Illumina BCL output using GenerateFastq of the Illumina HiSeq Analysis (HAS 2.2) Software.

Variant calling

The GATK data pre-processing workflow was used to generate analysis-ready alignments, including alignment, removing duplicate reads, local realignment and base quality recalibration. Specifically, raw sequence reads were aligned to the Human Reference Genome (NCBI Build 38) using the Burrows-Wheeler Aligner (BWA 0.7.12) and duplicate reads were removed using Picard.

Variant filtering

A series of filters were applied to keep only rare and functional SNVs and indels in our analysis. Only bi-allelic SNVs and indels were included. Genotypes with read depth < 3 were considered missing and long

indels (>10bp) were removed. Variants inside regions that tend to have high false positive rate of variant calling were excluded, including segmental duplications with greater than 96% similarity, low complexity regions, and tandem repeats. The coordinates of the segmental duplications were compiled from the segmental duplication regions obtained from the UCSC Genome Browser [1] and the Genome Aggregation Database (gnomAD)[2]. The coordinates of low complexity regions and tandem repeats were obtained from gnomAD and UCSC genome browser respectively. To keep only rare variants, we excluded any SNVs or indels with minor allele frequency (MAF) ≥ 0.5% in non-Finnish European population or MAF > 1% in any populations with more than 250 individuals from either exome or genome data of gnomAD. To obtain functional coding variants, we excluded non-exonic variants (except splicing variants), non-frameshift variants, synonymous variants, and nonsynonymous variants that were predicted to be deleterious by less than two out of the 12 prediction methods[3], including SIFT, Polyphen2_HDIV, Polyphen2_HVAR, LRT, MutationTaster, MutationAssessor, FATHMM, PROVEAN, MetaSVM, MetaLR, M-CAP, and fathmm-MKL. To obtain functional noncoding variants, we excluded nonsynonymous variants, splicing variants, frameshift variants, stopgain and stoploss variants, and further excluded any remaining variants that were predicted to be deleterious by less than three out of the six prediction methods, including CADD (PHRED-like scaled C-score ≥ 15), DANN (score ≥ 0.8), fathmm-XF (score > 0.5), LINSIGHT (score ≥ 0.2), funseq (score ≥ 2), and RegulomeDB (score ≤ 4). ANNOVAR[4] and WGSA[5] were used to facilitate these variant filtering steps. To assign a noncoding variant to its target gene, we utilized two genome-wide databases (GeneHancer[6] and BENGI[7]) with curated enhancer and target gene relationships. When a functional noncoding variant overlapped with an enhancer from the two databases, the target genes of the corresponding enhancer were assigned to the variant; otherwise, if the variant was within 10kb of a gene body, the target gene of the variant was the nearest gene. The noncoding variants without a target gene assignment were excluded from analysis.

Similar filters were also applied on SVs to keep rare functional SVs in analysis. We excluded SVs with ≥ 50% overlap with low complexity regions, tandem repeats, and segmental duplications with greater than 96% similarity. In addition, SVs with ≥ 50% overlap with common SVs (allele frequency ≥ 5% and sample size ≥ 50 in DGV[8] or gnomAD) were removed. When an SV overlapped with a gene body (with 1kb extension upstream), the gene was considered as the target gene of the SV; otherwise, if the SV overlapped with an enhancer in GeneHancer or BENGI (at least 50% of the SV overlap with the enhancer or 80% of the enhancer overlap with the SV), the enhancer's target genes were assigned to the SV. SVs without a target gene assignment were excluded from analysis.

DEG analysis in TCGA cohort

Batch-corrected, normalized mRNA gene expression were obtained from TCGA Pan-Cancer Atlas website (https://gdc.cancer.gov/about-data/publications/pancanatlas). We kept only primary breast and ovarian cancer samples that the corresponding patients were non-Hispanic whites, passed quality control, had tumor purity estimates ≥ 0.7 using the ESTIMATE method[9,10], and had germline WES data. DESeq2[11] was used to compare mRNA gene expression between *BRCA* carriers and non-carriers in breast and ovarian cancer separately. *BRCA* carriers were the patients carried pathogenic, likely pathogenic, or prioritized VUS germline variants in *BRCA1* or *BRCA2* gene based on germline WES variants from the TCGA PanCanAtlas Germline Working Group[12] (https://gdc.cancer.gov/about-data/publications/PanCanAtlas-Germline-AWG). Genes with adjusted p-values ≤ 0.05 from DESeq2 were considered DEGs.

References

1. Kent, W.J.; Sugnet, C.W.; Furey, T.S.; Roskin, K.M.; Pringle, T.H.; Zahler, A.M.; Haussler; David. The Human Genome Browser at UCSC. *Genome Research* **2002**, *12*, 996-1006, doi:10.1101/gr.229102.
2. Karczewski, K.J.; Francioli, L.C.; Tiao, G.; Cummings, B.B.; Alföldi, J.; Wang, Q.; Collins, R.L.; Laricchia, K.M.; Ganna, A.; Birnbaum, D.P.; et al. The mutational constraint spectrum quantified from variation in 141,456 humans. *Nature* **2020**, *581*, 434-443, doi:10.1038/s41586-020-2308-7.

3. Liu, X.; Jian, X.; Boerwinkle, E. dbNSFP: A lightweight database of human nonsynonymous SNPs and their functional predictions. *Human Mutation* **2011**, *32*, 894-899, doi:10.1002/humu.21517.
4. Wang, K.; Li, M.; Hakonarson, H. ANNOVAR: functional annotation of genetic variants from high-throughput sequencing data. *Nucleic Acids Research* **2010**, *38*, e164, doi:10.1093/nar/gkq603.
5. Liu, X.; White, S.; Peng, B.; Johnson, A.D.; Brody, J.A.; Li, A.H.; Huang, Z.; Carroll, A.; Wei, P.; Gibbs, R.; et al. WGSA: an annotation pipeline for human genome sequencing studies. *Journal of Medical Genetics* **2016**, *53*, 111-112, doi:10.1136/jmedgenet-2015-103423.
6. Fishilevich, S.; Nudel, R.; Rappaport, N.; Hadar, R.; Plaschkes, I.; Iny Stein, T.; Rosen, N.; Kohn, A.; Twik, M.; Safran, M.; et al. GeneHancer: genome-wide integration of enhancers and target genes in GeneCards. *Database (Oxford)* **2017**, *2017*, doi:10.1093/database/bax028.
7. Moore, J.E.; Pratt, H.E.; Purcaro, M.J.; Weng, Z. A curated benchmark of enhancer-gene interactions for evaluating enhancer-target gene prediction methods. *Genome Biol* **2020**, *21*, 17, doi:10.1186/s13059-019-1924-8.
8. MacDonald, J.R.; Ziman, R.; Yuen, R.K.; Feuk, L.; Scherer, S.W. The Database of Genomic Variants: a curated collection of structural variation in the human genome. *Nucleic Acids Res* **2014**, *42*, D986-992, doi:10.1093/nar/gkt958.
9. Aran, D.; Sirota, M.; Butte, A.J. Systematic pan-cancer analysis of tumour purity. *Nature communications* **2015**, *6*, 8971, doi:10.1038/ncomms9971.
10. Yoshihara, K.; Shahmoradgoli, M.; Martínez, E.; Vegesna, R.; Kim, H.; Torres-Garcia, W.; Treviño, V.; Shen, H.; Laird, P.W.; Levine, D.A.; et al. Inferring tumour purity and stromal and immune cell admixture from expression data. *Nature communications* **2013**, *4*, 2612, doi:10.1038/ncomms3612.
11. Love, M.I.; Huber, W.; Anders, S. Moderated estimation of fold change and dispersion for RNA-seq data with DESeq2. *Genome Biology* **2014**, *15*, 550, doi:10.1186/s13059-014-0550-8.
12. Huang, K.L.; Mashl, R.J.; Wu, Y.; Ritter, D.I.; Wang, J.; Oh, C.; Paczkowska, M.; Reynolds, S.; Wyczalkowski, M.A.; Oak, N.; et al. Pathogenic Germline Variants in 10,389 Adult Cancers. *Cell* **2018**, *173*, 355-370.e314, doi:10.1016/j.cell.2018.03.039.

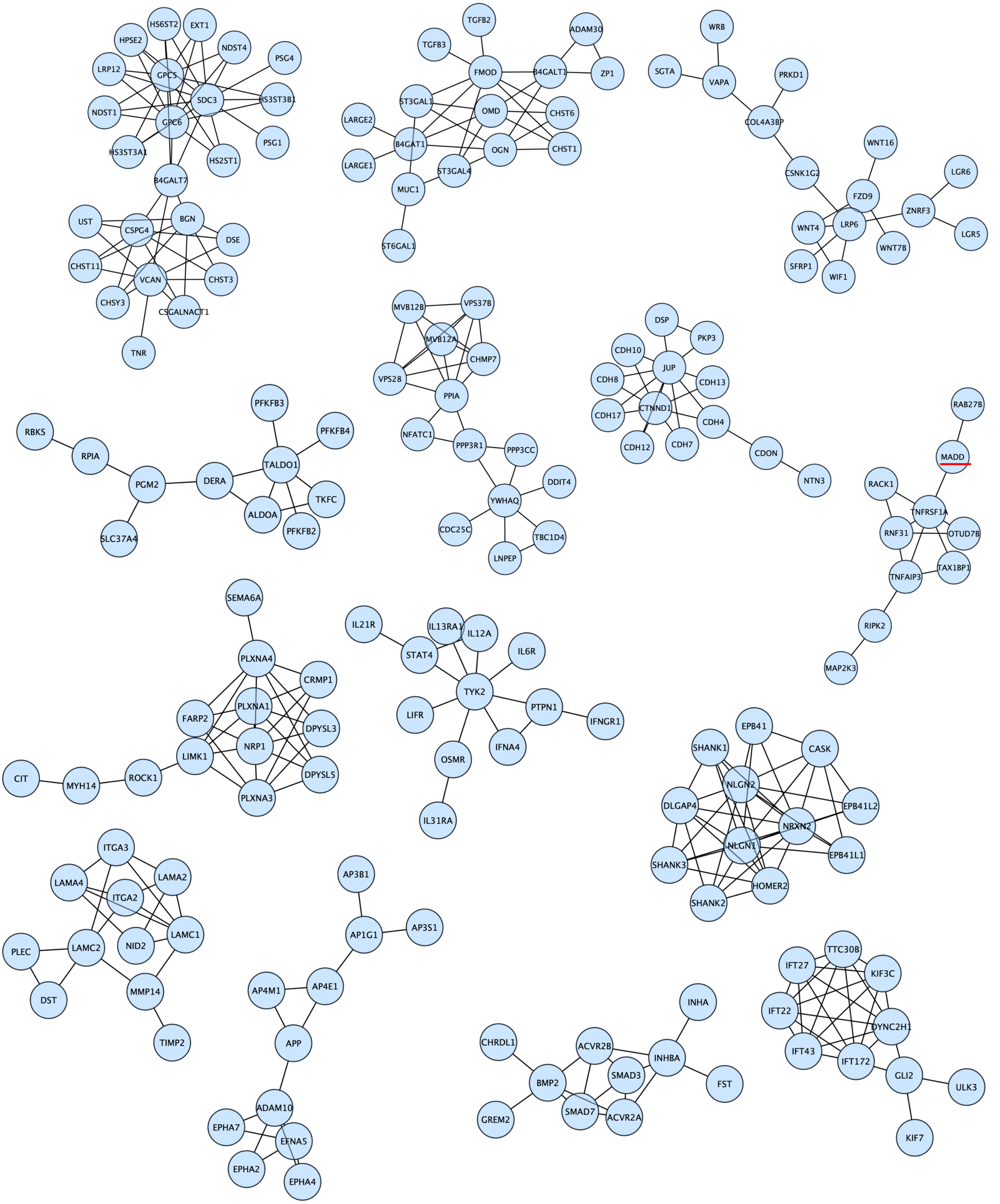

Figure S1. The remaining 14 gene sub-networks significantly altered in *BRCA* carriers. Genes that were significantly highly mutated in *BRCA* carriers (Table 1) were highlighted by red underscores.

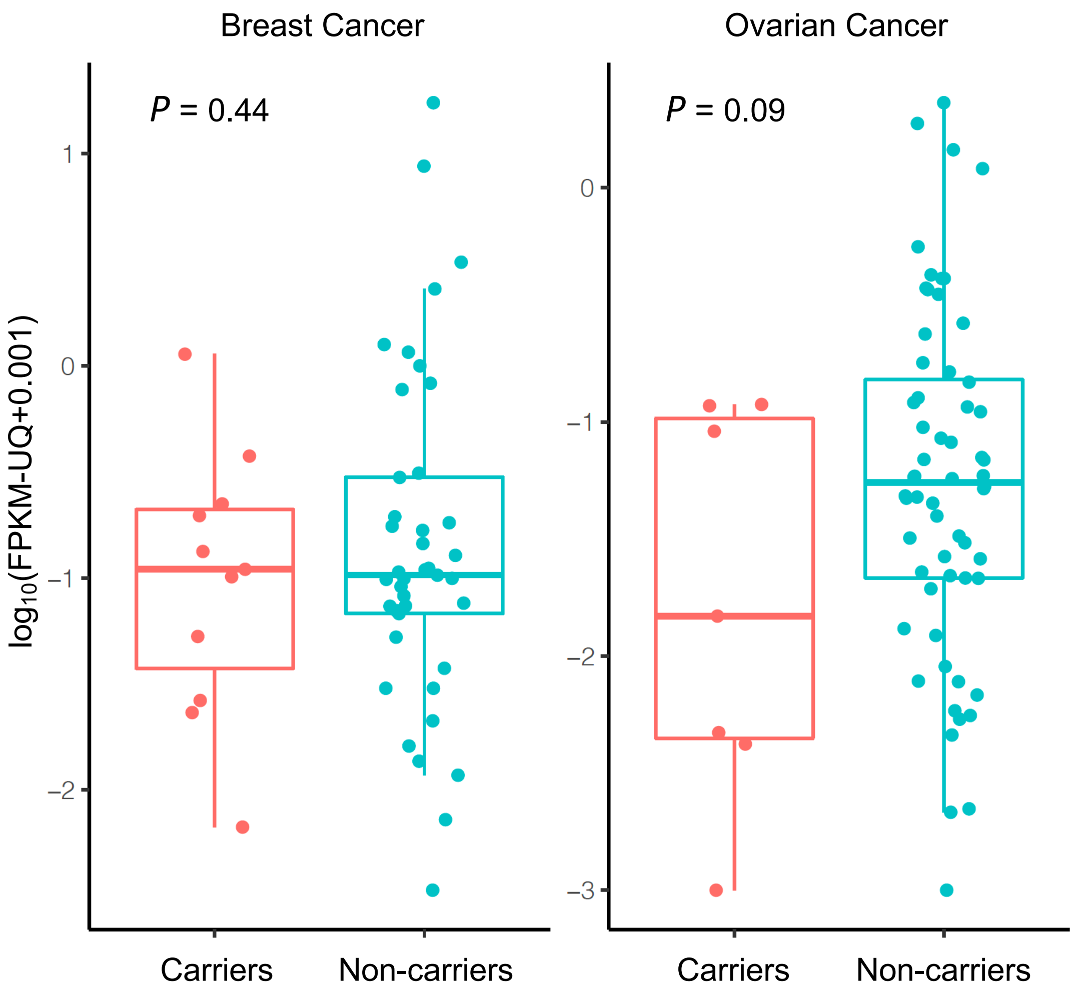

Figure S2. *PPARGC1A* expression between carriers of non-coding mutations and non-carriers in PCAWG. Gene expression levels (FPKM-UQ) were estimated using the FPKM metric, based on alignments from the TopHat and STAR algorithms and normalised with the Upper Quartile method. FPKM, fragments per kilobase of transcript per million mapped reads. P-values were calculated using one-sided Wilcoxon rank sum test between 11 carriers and 41 non-carriers in breast cancer and between 7 carriers and 60 non-carriers in ovarian cancer.

Table S1. Characteristics of the discovery cohort.

Characteristic	N (%)			
	Hereditary OC		Sporadic OC	
Age at diagnosis, years range (median)	29-81 (53)*		39-86 (65)	
Histology				
Serous	30	(61.22%)	13	(76.47%)
Endometrioid	3	(6.12%)	1	(5.88%)
Clear Cell	2	(4.08%)	1	(5.88%)
Other	4	(8.16%)	2	(11.76%)
Unknown	10	(20.41%)	0	(0%)
Stage				
I	10	(20.41%)	1	(5.88%)
II	3	(6.12%)	0	(0%)
III	21	(42.86%)	14	(82.35%)
IV	1	(2.04%)	1	(5.88%)
Unknown	14	(28.57%)	1	(5.88%)
Grade				
1	1	(2.04%)	0	(0%)
2	4	(8.16%)	2	(11.76%)
3	31	(63.27%)	12	(70.59%)
4	0	(0%)	3	(17.65%)
Unknown	13	(26.53%)	0	(0%)
*: age at diagnosis information was missing in five patients.				

Table S2. The sequencing coverage and quality statistics of whole-genome sequenced hereditary OC patients from FOCR.

Sample ID	Total number of sequenced reads	Total number of uniquely mapped nonduplicate reads*	Total number of covered bases	Median coverage per base§	Percentage of targeted bases with coverage ≥10§
1	935302026	811972475	122607843725	40	99.2
2	935845222	825200823	124605324273	41	99.2
3	952160346	825372842	124631299142	41	99.2
4	924918982	805201697	121585456247	40	99.2
5	953623992	826646366	124823601266	41	99.2
6	960580044	827374141	124933495291	41	99.2
7	957323482	835352293	126138196243	42	99.2
8	938351662	820663671	123920214321	41	99.2
9	966735394	845639373	127691545323	42	99.2
10	952528672	842860146	127271882046	42	99.2
11	965301722	863054397	130321213947	43	99.2
12	1004767130	872690388	131776248588	43	99.2
13	971041556	832094850	125646322350	41	99.2
14	977418806	827298517	124922076067	41	99.2
15	952268794	799051383	120656758833	40	99.2
16	1023682316	820257796	123858927196	41	99.2
17	997514272	773513766	116800578666	38	99.2
18	1016513828	758110908	114474747108	37	99.2
19	977898390	727738749	109888551099	36	99.2
20	1028641558	753336200	113753766200	37	99.2
21	999786454	753495441	113777811591	37	99.2
22	1020260814	775432313	117090279263	38	99.2
23	1026192418	740165438	111764981138	37	99.2
24	933432630	793266433	119783231383	39	99.2
25	940787544	799975750	120796338250	39	99.2
26	949464588	813810106	122885326006	40	99.2
27	953957168	817090145	123380611895	40	99.2
28	974735664	828393115	125087360365	41	99.2
29	950416164	814205667	122945055717	40	99.2
30	951035190	804024979	121407771829	40	99.2
31	984864632	838782561	126656166711	42	99.2
32	966504858	821011212	123972693012	41	99.2
33	985484154	829413023	125241366473	41	99.2
34	977598918	820387012	123878438812	41	99.2
35	998740086	831832417	125606694967	41	99.2

36	976671768	833014809	125785236159	41	99.2
37	976850424	829448693	125246752643	41	99.3
38	976974002	827698786	124982516686	41	99.2
39	1027895158	811532484	122541405084	40	99.2
40	1006287568	841156488	127014629688	42	99.2
41	990897126	843824181	127417451331	42	99.3
42	1022699462	821738620	124082531620	41	99.2
43	1025707330	822024113	124125641063	41	99.3
44	1005593764	841328422	127040591722	42	99.2
45	1012190146	823036766	124278551666	41	99.2
46	995567930	818222377	123551578927	41	99.2
47	992431524	848665111	128148431761	42	99.2
48	912619224	765797018	115635349718	38	99.2
49	958684552	836355625	126289699375	41	99.2

*: reference genome GRCh38 was used.
§: targeted bases refer to all gene regions.

Table S3. The sequencing coverage and quality statistics of whole-genome sequenced sporadic OC patients.

Sample ID	Total number of sequenced reads	Total number of uniquely mapped nonduplicate reads*	Total number of covered bases	Median coverage per base§	Percentage of targeted bases with coverage ≥10§
1	969170002	797176812	120373698612	40	99.2
2	910238894	781291656	117975040056	39	99.2
3	941147180	764931844	115504708444	38	99.2
4	949072664	775158796	117048978196	39	99.2
5	895076510	766231293	115700925243	38	99.2
6	832089548	739110356	111605663756	37	99.1
7	912144608	779259299	117668154149	39	99.2
8	827628702	754793416	113973805816	38	99.1
9	881814706	773450843	116791077293	38	99.2
10	906874704	777439275	117393330525	39	99.2
11	922243332	795194931	120074434581	40	99.2
12	889666784	777832140	117452653140	39	99.2
13	884984776	773047027	116730101077	38	99.1
14	828659944	757618161	114400342311	38	99.2
15	880668120	779939678	117770891378	39	99.2
16	764612670	713541678	107744793378	35	99.1
17	800175668	742870367	112173425417	37	99.1

*: reference genome GRCh38 was used.
§: targeted bases refer to all gene regions.

Table S4. The *PPARGC1A* variants included in our analyses*.

Chr	Position§	Reference allele	Alternative allele	Alternative allele frequency§	Amino acid change†	Prediction programs‡	Observed cohort
4	23770078	C	A	3.26E-03		CADD, LINSIGHT, RegulomeDB	Discovery, PCAWG
4	23773095	A	T			CADD, DANN, fathmm-XF, LINSIGHT	PCAWG
4	23789494	C	T	0		CADD, fathmm-XF, LINSIGHT	PCAWG
4	23790313	G	C	9.33E-04		CADD, DANN, fathmm-XF, LINSIGHT	Discovery
4	23792427	G	A	2.80E-03		DANN, fathmm-XF, LINSIGHT	Discovery, PCAWG
4	23792491	G	A	0		DANN, fathmm-XF, LINSIGHT	PCAWG
4	23793325	C	T	6.67E-05		DANN, fathmm-XF, LINSIGHT	PCAWG
4	23794025	A	G	2.00E-04		DANN, fathmm-XF, LINSIGHT	PCAWG
4	23795705	A	G	5.34E-04		DANN, fathmm-XF, LINSIGHT	Discovery
4	23795778	T	G			DANN, fathmm-XF, LINSIGHT	PCAWG
4	23801814	T	C		T737A	Polyphen2_HDIV, Polyphen2_HVAR, LRT, MutationTaster, fathmm-MKL	PCAWG
4	23802297	G	A		R690W	SIFT, Polyphen2_HDIV, Polyphen2_HVAR, LRT, MutationTaster, MutationAssessor, PROVEAN, M-CAP, fathmm-MKL	PCAWG
4	23806392	A	G			CADD, DANN, fathmm-XF, LINSIGHT	PCAWG
4	23814572	G	A	2.00E-04	A304V	LRT, MutationTaster, MutationAssessor, fathmm-MKL	PCAWG
4	23818215	T	G	4.87E-03		CADD, fathmm-XF, LINSIGHT, RegulomeDB	Discovery, PCAWG
4	23820689	A	G			CADD, DANN, LINSIGHT	Discovery
4	23820712	T	C			CADD, DANN, LINSIGHT	PCAWG
4	23824951	T	A			CADD, DANN, fathmm-XF, LINSIGHT	PCAWG
4	23829109	G	A	9.99E-04		CADD, fathmm-XF, LINSIGHT	PCAWG
4	23832301	T	G	2.67E-04		CADD, DANN, LINSIGHT	PCAWG

4	23845835	A	G	0		CADD, DANN, LINSIGHT	PCAWG
4	23856582	A	G	2.27E-03		CADD, DANN, LINSIGHT	PCAWG
4	23857558	C	T	1.07E-03		CADD, DANN, RegulomeDB	PCAWG
4	23866718	C	T			CADD, DANN, LINSIGHT	PCAWG
4	23871103	T	C	2.00E-04		CADD, DANN, fathmm-XF, LINSIGHT	PCAWG
4	23874718	T	C			DANN, LINSIGHT, RegulomeDB	PCAWG
4	23874735	A	C	3.33E-04		fathmm-XF, LINSIGHT, RegulomeDB	PCAWG
4	23876219	C	T	3.33E-04		CADD, fathmm-XF, LINSIGHT	PCAWG
4	23876269	G	C			DANN, fathmm-XF, LINSIGHT	Discovery
4	23877705	C	T	1.47E-03		CADD, LINSIGHT, RegulomeDB	PCAWG
4	23878904	C	T			CADD, fathmm-XF, LINSIGHT, funseq	Discovery
4	23879099	A	T			CADD, DANN, LINSIGHT, funseq	PCAWG
4	23884497	A	G			fathmm-XF, LINSIGHT, RegulomeDB	PCAWG
4	23884765	G	A	9.00E-04	S74L	SIFT, LRT, MutationTaster, MutationAssessor, fathmm-MKL	Discovery, PCAWG
4	23888508	C	G	1.33E-04		fathmm-XF, LINSIGHT, RegulomeDB	PCAWG
4	23888511	T	G	1.33E-04		LINSIGHT, funseq, RegulomeDB	PCAWG
4	23890008	A	C			DANN, fathmm-XF, LINSIGHT, RegulomeDB	PCAWG
4	23890048	G	T	1.47E-03		DANN, fathmm-XF, LINSIGHT, funseq, RegulomeDB	Discovery, PCAWG
4	23890401	A	T	6.68E-05		CADD, DANN, fathmm-XF, LINSIGHT, funseq	PCAWG
4	23892007	A	G	3.00E-03		CADD, DANN, fathmm-XF, LINSIGHT	PCAWG
4	23892109	A	T			DANN, fathmm-XF, funseq	PCAWG
4	23892769	G	A	0		CADD, fathmm-XF, LINSIGHT	PCAWG
4	23894203	A	G	0		CADD, DANN, LINSIGHT	PCAWG
4	23894291	A	C			CADD, DANN, LINSIGHT	PCAWG
4	23903060	A	G			CADD, DANN, LINSIGHT	PCAWG

4	23904290	C	G	1.33E-04		DANN, fathmm-XF, LINSIGHT, RegulomeDB	PCAWG
4	23905144	A	G			CADD, fathmm-XF, LINSIGHT	Discovery
4	23905150	A	G			CADD, fathmm-XF, LINSIGHT	PCAWG
4	23930998	G	A	1.33E-04		DANN, LINSIGHT, RegulomeDB	PCAWG
4	23931008	T	C	2.00E-03		fathmm-XF, LINSIGHT, RegulomeDB	PCAWG
4	23931055	G	A	2.08E-03		DANN, fathmm-XF, LINSIGHT, RegulomeDB	PCAWG
4	23931165	G	C			DANN, fathmm-XF, LINSIGHT, RegulomeDB	Discovery
4	23934989	A	G			CADD, DANN, LINSIGHT, RegulomeDB	PCAWG
4	23934996	T	G	2.87E-03		CADD, DANN, LINSIGHT, funseq, RegulomeDB	Discovery
4	23935004	C	G	1.47E-03		CADD, LINSIGHT, RegulomeDB	PCAWG
4	24219261	A	G	2.66E-04		DANN, LINSIGHT, RegulomeDB	Discovery

[*]: only rare and potentially functional variants were included in our analysis (Supplementary Methods).
[š]: based on genome build 38.
[§]: alternative allele frequency in gnomAD non-Finish European genomes. Variants not present in the gnomAD whole genomes annotation database used by ANNOVAR or WGSA were left blank.
[†]: amino acid change is based on transcript uc003gqs.3. Blank for non-coding variants.
[‡]: the prediction programs that predicted the corresponding variant to be functional.

Table S5: The pathways significantly enriched in the genes within the largest gene sub-network significantly altered in *BRCA* carriers.

ID	Description	GeneRatio	BgRatio	pvalue	p.adjust	qvalue	geneID	Count
R-HSA-381340	Transcriptional regulation of white adipocyte differentiation	10/47	84/10654	2.19E-12	3.56E-10	2.03E-10	CEBPB/EBF1/KLF4/KLF5/MED26/MED8/PPARG/PPARGC1A/ZNF467/ZNF638	10
R-HSA-9013508	NOTCH3 Intracellular Domain Regulates Transcription	7/47	25/10654	9.23E-12	7.53E-10	4.28E-10	HEY1/HEYL/MAML1/MAML3/PBX1/PTCRA/RBPJ	7
R-HSA-400253	Circadian Clock	8/47	70/10654	5.9E-10	3.21E-08	1.82E-08	CLOCK/MEF2C/MEF2D/NPAS2/NRIP1/PER2/PPARGC1A/RORA	8
R-HSA-2122947	NOTCH1 Intracellular Domain Regulates Transcription	7/47	47/10654	1.12E-09	4.58E-08	2.6E-08	HEY1/HEYL/MAML1/MAML3/RBPJ/TLE3/TLE4	7
R-HSA-9012852	Signaling by NOTCH3	7/47	49/10654	1.52E-09	4.97E-08	2.82E-08	HEY1/HEYL/MAML1/MAML3/PBX1/PTCRA/RBPJ	7
R-HSA-2151201	Transcriptional activation of mitochondrial biogenesis	7/47	56/10654	4.02E-09	1.09E-07	6.21E-08	MEF2C/MEF2D/POLRMT/PPARGC1A/PPRC1/TFB1M/TFB2M	7
R-HSA-1980143	Signaling by NOTCH1	7/47	73/10654	2.67E-08	6.22E-07	3.54E-07	HEY1/HEYL/MAML1/MAML3/RBPJ/TLE3/TLE4	7
R-HSA-3108232	SUMO E3 ligases SUMOylate target proteins	9/47	182/10654	7.92E-08	1.61E-06	9.17E-07	BRCA1/NRIP1/PIAS1/PPARG/PPARGC1A/RARA/RORA/SATB2/XRCC4	9
R-HSA-2990846	SUMOylation	9/47	188/10654	1.05E-07	1.9E-06	1.08E-06	BRCA1/NRIP1/PIAS1/PPARG/PPARGC1A/RARA/RORA/SATB2/XRCC4	9
R-HSA-1592230	Mitochondrial biogenesis	7/47	95/10654	1.69E-07	2.75E-06	1.56E-06	MEF2C/MEF2D/POLRMT/PPARGC1A/PPRC1/TFB1M/TFB2M	7

R-HSA-157118	Signaling by NOTCH	9/47	235/10654	6.96E-07	9.55E-06	5.43E-06	HEY1/HEYL/MAML1/MAML3/PBX1/PTCRA/RBPJ/TLE3/TLE4	9
R-HSA-1989781	PPARA activates gene expression	7/47	117/10654	7.03E-07	9.55E-06	5.43E-06	CLOCK/MED26/MED8/NPAS2/PPARG/PPARGC1A/RORA	7
R-HSA-400206	Regulation of lipid metabolism by Peroxisome proliferator-activated receptor alpha (PPARalpha)	7/47	119/10654	7.89E-07	9.9E-06	5.62E-06	CLOCK/MED26/MED8/NPAS2/PPARG/PPARGC1A/RORA	7
R-HSA-9013695	NOTCH4 Intracellular Domain Regulates Transcription	4/47	20/10654	1.53E-06	1.78E-05	1.01E-05	HEY1/MAML1/MAML3/RBPJ	4
R-HSA-2644602	Signaling by NOTCH1 PEST Domain Mutants in Cancer	5/47	58/10654	5.17E-06	4.43E-05	2.52E-05	HEY1/HEYL/MAML1/MAML3/RBPJ	5
R-HSA-2644603	Signaling by NOTCH1 in Cancer	5/47	58/10654	5.17E-06	4.43E-05	2.52E-05	HEY1/HEYL/MAML1/MAML3/RBPJ	5
R-HSA-2644606	Constitutive Signaling by NOTCH1 PEST Domain Mutants	5/47	58/10654	5.17E-06	4.43E-05	2.52E-05	HEY1/HEYL/MAML1/MAML3/RBPJ	5
R-HSA-2894858	Signaling by NOTCH1 HD+PEST Domain Mutants in Cancer	5/47	58/10654	5.17E-06	4.43E-05	2.52E-05	HEY1/HEYL/MAML1/MAML3/RBPJ	5
R-HSA-2894862	Constitutive Signaling by NOTCH1 HD+PEST Domain Mutants	5/47	58/10654	5.17E-06	4.43E-05	2.52E-05	HEY1/HEYL/MAML1/MAML3/RBPJ	5
R-HSA-5693532	DNA Double-Strand Break Repair	7/47	166/10654	7.31E-06	5.96E-05	3.39E-05	BRCA1/BRCA2/GEN1/HUS1/PRKDC/TOPBP1/XRCC4	7
R-HSA-4090294	SUMOylation of intracellular receptors	4/47	30/10654	8.38E-06	6.5E-05	3.69E-05	PIAS1/PPARG/RARA/RORA	4

R-HSA-5685942	HDR through Homologous Recombination (HRR)	5/47	67/10654	1.06E-05	7.83E-05	4.45E-05	BRCA1/BRCA2/GEN1/HUS1/TOPBP1	5
R-HSA-2197563	NOTCH2 intracellular domain regulates transcription	3/47	12/10654	1.72E-05	0.000117	6.65E-05	MAML1/MAML3/RBPJ	3
R-HSA-4641265	Repression of WNT target genes	3/47	12/10654	1.72E-05	0.000117	6.65E-05	TCF7L2/TLE3/TLE4	3
R-HSA-8939243	RUNX1 interacts with co-factors whose precise effect on RUNX1 targets is not known	4/47	38/10654	2.2E-05	0.000143	8.15E-05	ARID1B/PBRM1/SMARCA4/SMARCE1	4
R-HSA-5693616	Presynaptic phase of homologous DNA pairing and strand exchange	4/47	39/10654	2.44E-05	0.000153	8.7E-05	BRCA1/BRCA2/HUS1/TOPBP1	4
R-HSA-8941856	RUNX3 regulates NOTCH signaling	3/47	14/10654	2.83E-05	0.000171	9.71E-05	MAML1/MAML3/RBPJ	3
R-HSA-3769402	Deactivation of the beta-catenin transactivating complex	4/47	42/10654	3.29E-05	0.000185	0.000105	BCL9/TCF7L2/TLE3/TLE4	4
R-HSA-5693579	Homologous DNA Pairing and Strand Exchange	4/47	42/10654	3.29E-05	0.000185	0.000105	BRCA1/BRCA2/HUS1/TOPBP1	4
R-HSA-210744	Regulation of gene expression in late stage (branching morphogenesis) pancreatic bud precursor cells	3/47	16/10654	4.33E-05	0.000235	0.000134	MAML1/MAML3/RBPJ	3
R-HSA-201722	Formation of the beta-catenin:TCF	5/47	91/10654	4.7E-05	0.000247	0.000141	BCL9/SMARCA4/TCF7L2/TLE3/TLE4	5

	transactivating complex							
R-HSA-73894	DNA Repair	8/47	334/10654	9.27E-05	0.000472	0.000268	BRCA1/BRCA2/GEN1/HUS1/PIAS1/PRKDC/TOPBP1/XRCC4	8
R-HSA-350054	Notch-HLH transcription pathway	3/47	28/10654	0.000244	0.001205	0.000685	MAML1/MAML3/RBPJ	3
R-HSA-5693567	HDR through Homologous Recombination (HRR) or Single Strand Annealing (SSA)	5/47	132/10654	0.000273	0.001311	0.000745	BRCA1/BRCA2/GEN1/HUS1/TOPBP1	5
R-HSA-1852241	Organelle biogenesis and maintenance	7/47	296/10654	0.000288	0.001343	0.000763	MEF2C/MEF2D/POLRMT/PPARGC1A/PPRC1/TFB1M/TFB2M	7
R-HSA-525793	Myogenesis	3/47	30/10654	0.000301	0.001361	0.000773	MEF2C/MEF2D/TCF4	3
R-HSA-5693538	Homology Directed Repair	5/47	138/10654	0.000336	0.00148	0.000841	BRCA1/BRCA2/GEN1/HUS1/TOPBP1	5
R-HSA-3214858	RMTs methylate histone arginines	4/47	79/10654	0.000392	0.00163	0.000927	ARID1B/PBRM1/SMARCA4/SMARCE1	4
R-HSA-1980145	Signaling by NOTCH2	3/47	33/10654	0.0004	0.00163	0.000927	MAML1/MAML3/RBPJ	3
R-HSA-5693568	Resolution of D-loop Structures through Holliday Junction Intermediates	3/47	33/10654	0.0004	0.00163	0.000927	BRCA1/BRCA2/GEN1	3
R-HSA-5693537	Resolution of D-Loop Structures	3/47	34/10654	0.000437	0.001739	0.000988	BRCA1/BRCA2/GEN1	3
R-HSA-9013694	Signaling by NOTCH4	4/47	82/10654	0.000452	0.001754	0.000997	HEY1/MAML1/MAML3/RBPJ	4
R-HSA-5685938	HDR through Single Strand Annealing (SSA)	3/47	37/10654	0.000563	0.002133	0.001212	BRCA1/HUS1/TOPBP1	3

R-HSA-186712	Regulation of beta-cell development	3/47	42/10654	0.000819	0.002973	0.001689	MAML1/MAML3/RBPJ	3
R-HSA-8878159	Transcriptional regulation by RUNX3	4/47	96/10654	0.000821	0.002973	0.001689	MAML1/MAML3/RBPJ/TCF7L2	4
R-HSA-3899300	SUMOylation of transcription cofactors	3/47	45/10654	0.001003	0.003553	0.002019	NRIP1/PIAS1/PPARGC1A	3
R-HSA-383280	Nuclear Receptor transcription pathway	3/47	52/10654	0.001528	0.0053	0.003012	PPARG/RARA/RORA	3
R-HSA-5693571	Nonhomologous End-Joining (NHEJ)	3/47	69/10654	0.003439	0.011573	0.006577	BRCA1/PRKDC/XRCC4	3
R-HSA-201681	TCF dependent signaling in response to WNT	5/47	233/10654	0.003479	0.011573	0.006577	BCL9/SMARCA4/TCF7L2/TLE3/TLE4	5
R-HSA-3108214	SUMOylation of DNA damage response and repair proteins	3/47	77/10654	0.004684	0.015271	0.008678	BRCA1/PIAS1/XRCC4	3
R-HSA-452723	Transcriptional regulation of pluripotent stem cells	2/47	24/10654	0.004942	0.015492	0.008804	KLF4/PBX1	2
R-HSA-8940973	RUNX2 regulates osteoblast differentiation	2/47	24/10654	0.004942	0.015492	0.008804	HEY1/SATB2	2
R-HSA-195253	Degradation of beta-catenin by the destruction complex	3/47	82/10654	0.005585	0.017175	0.009761	TCF7L2/TLE3/TLE4	3
R-HSA-5693554	Resolution of D-loop Structures through Synthesis-Dependent Strand Annealing (SDSA)	2/47	26/10654	0.005787	0.017468	0.009927	BRCA1/BRCA2	2

R-HSA-5663202	Diseases of signal transduction	6/47	377/10654	0.005942	0.017611	0.010008	HEY1/HEYL/MAML1/MAML3/RBPJ/TCF7L2	6
R-HSA-1368108	BMAL1:CLOCK,NPAS2 activates circadian gene expression	2/47	27/10654	0.006232	0.018141	0.010309	CLOCK/NPAS2	2
R-HSA-3247509	Chromatin modifying enzymes	5/47	274/10654	0.006887	0.019354	0.010999	ARID1B/CLOCK/PBRM1/SMARCA4/SMARCE1	5
R-HSA-4839726	Chromatin organization	5/47	274/10654	0.006887	0.019354	0.010999	ARID1B/CLOCK/PBRM1/SMARCA4/SMARCE1	5
R-HSA-9616222	Transcriptional regulation of granulopoiesis	3/47	90/10654	0.007229	0.019971	0.011349	CEBPB/KLF5/RARA	3
R-HSA-6804756	Regulation of TP53 Activity through Phosphorylation	3/47	92/10654	0.00768	0.020864	0.011857	BRCA1/HUS1/TOPBP1	3
R-HSA-1912408	Pre-NOTCH Transcription and Translation	3/47	93/10654	0.007912	0.021141	0.012014	MAML1/MAML3/RBPJ	3
R-HSA-69473	G2/M DNA damage checkpoint	3/47	95/10654	0.008388	0.022051	0.012531	BRCA1/HUS1/TOPBP1	3
R-HSA-8941326	RUNX2 regulates bone development	2/47	32/10654	0.008685	0.02247	0.01277	HEY1/SATB2	2
R-HSA-5693607	Processing of DNA double-strand break ends	3/47	98/10654	0.009132	0.023259	0.013218	BRCA1/HUS1/TOPBP1	3
R-HSA-1912422	Pre-NOTCH Expression and Processing	3/47	109/10654	0.012188	0.030564	0.017369	MAML1/MAML3/RBPJ	3
R-HSA-195721	Signaling by WNT	5/47	331/10654	0.014803	0.036559	0.020776	BCL9/SMARCA4/TCF7L2/TLE3/TLE4	5
R-HSA-8878166	Transcriptional regulation by RUNX2	3/47	121/10654	0.016118	0.039211	0.022283	HEY1/PPARGC1A/SATB2	3
R-HSA-8878171	Transcriptional regulation by RUNX1	4/47	239/10654	0.020729	0.049688	0.028238	ARID1B/PBRM1/SMARCA4/SMARCE1	4

Table S6. Comparison of *PPARGC1A* mutation frequency between *BRCA* carriers and non-carriers in the validation cohorts.

Cancer type	Project	# of *BRCA* carriers	# of non-carriers	# and fraction of *BRCA* carriers with *PPARGC1A* mutations		# and fraction of non-carriers with *PPARGC1A* mutations		p-value*
Ovarian	TCGA	4	30	1	0.25	4	0.13	0.49
	ICGC	13	44	0	0	5	0.11	1.00
	Combined	17	74	1	0.06	9	0.12	0.89
Breast	TCGA	4	48	2	0.5	9	0.19	0.19
	ICGC	23	81	5	0.22	14	0.17	0.41
	Combined	27	129	7	0.26	23	0.18	0.24

*: raw p-value from one-sided Fisher's exact test (H_a: gene mutation frequency in *BRCA* carriers ≥ the frequency in non-carriers).

Table S7. Comparison of *PBX1* mutation frequency between *BRCA* carriers and non-carriers in the validation cohorts.

Cancer type	Project	# of *BRCA* carriers	# of non-carriers	# and fraction of *BRCA* carriers with *PBX1* mutations		# and fraction of non-carriers with *PBX1* mutations		p-value*
Ovarian	TCGA	4	30	1	0.25	8	0.27	
	ICGC	13	44	2	0.15	10	0.23	
	Combined	17	74	3	0.18	18	0.24	0.82
Breast	TCGA	4	48	1	0.25	13	0.27	
	ICGC	23	81	5	0.22	15	0.19	
	Combined	27	129	6	0.22	28	0.22	0.57

*: raw p-value from one-sided Fisher's exact test (H_a: gene mutation frequency in *BRCA* carriers ≥ the frequency in non-carriers).

Table S8. Linear regression between cancer age onset and gene mutation status (*BRCA*, *PPARGC1A*) in PCAWG*.

Cancer type	*BRCA* carrier		*PPARGC1A* carrier		Interaction		Sample size§			
	beta	p	beta	p	beta	p	*BRCA+ PPARGC1A+*	*BRCA+ PPARGC1A-*	*BRCA- PPARGC1A+*	*BRCA- PPARGC1A-*
Breast	2.65	0.45	3.29	0.32	-15.46	**0.03**	7	18	21	97
Ovarian	-3.67	0.18	2.63	0.45	6.12	0.56	1	16	9	65
Combined	0.12	0.96	2.45	0.33	-12.03	**0.03**	8	34	30	162

*: A linear regression model was used to associate age onset of breast or ovarian cancer with *BRCA* status, *PPARGC1A* mutation status, and their interaction term. Significant p-values (≤ 0.05) were in bold.

§: The number of patients by *BRCA* status and *PPARGC1A* mutation status: patients carried both *BRCA* mutations and *PPARGC1A* mutations (*BRCA*+ *PPARGC1A*+), patients carried *BRCA* mutations but no *PPARGC1A* mutations (*BRCA*+ *PPARGC1A*-), patients carried *PPARGC1A* mutations but no BRCA mutations (*BRCA*- *PPARGC1A*+), and patients carried neither *BRCA* mutations nor *PPARGC1A* mutations (*BRCA*- *PPARGC1A*-).

Table S9. Linear regression between cancer age onset and gene mutation status (*BRCA1*, *BRCA2, PPARGC1A*) in PCAWG*.

Cancer type	*BRCA1* carrier		*BRCA2* carrier		*PPARGC1A* carrier		Interaction between *BRCA1* and *PPARGC1A*		Interaction between *BRCA2* and *PPARGC1A*	
	beta	p	beta	p	beta	p	beta	p	beta	p
Breast	2.49	0.67	2.74	0.52	3.29	0.32	-16.29	0.09	-14.21	0.14
Ovarian	-5.65	0.08	0.68	0.88	2.63	0.45	NA	NA	1.77	0.87
Combined	-1.20	0.71	1.44	0.65	2.45	0.33	-14.33	0.06	-9.73	0.19

*: A linear regression model was used to associate age onset of breast or ovarian cancer with *BRCA1* status, *BRCA2* status and *PPARGC1A* mutation status, and their interaction terms. Significant p-values (≤ 0.05) were in bold.
